# Anatomy

**Published:** 1859-09

**Authors:** 


### ANATOMY.

THE STRUCTURE OF THE ULTIMATE AIR-TUBES, AND THE DISTRIBUTION OF THE BLOOD-VESSELS OF THE HUMAN LUNG. A paper read before the Royal Society of London, by *A. T. H. Waters, Esq.*, of Liverpool.—The bronchial tubes terminate in a dilatation, into which open a number of cavities to which various names have been given, but which the author proposes to call air-sacs. The air-sacs connected with a terminable bronchial twig, with their vessels, etc., constitute a lobulette. The lobulette consists of from six to twelve air-sacs; the latter are somewhat elongated cavities, lying side by side in the lobulette, and separated from each other by thin walls; in shape they are polygonal, from mutual pressure of their parietes. They all communicate with the dilated extremity of the bronchial tube, which forms the common mouth or centre of all the sacs; they have no lateral orifices of communication with each other; they often divide or give off other sacs; the air-sacs of one lobulette do not communicate with those of another. The walls of the air-sacs are covered by a number of small, shallow, cup-like depressions, separated from each other by partial septa. These depressions, or alveoli, are very numerous; their number varies in different air-sacs, from eight to twenty. The lobulettes are supported externally by the pleura, but within the lung, in part by the bronchial tubes and blood-vessels. The membrane forming the walls of the air-sacs in a lung inflated and dried is very transparent; it constitutes, by its projection toward the centre of the sacs, the septa of the alveoli. Each lobulette is distinct and separate from those which surround it. The separation may be sometimes seen in the inflated infants' lung, but the observation of the fœtal lung affords the best proof of it. The author alluded to investigations he had made on the lungs of fœtuses, which had confirmed the view he had taken of the arrangement of the ultimate pulmonary tissue, and of the separation between the lobulettes. The air-sacs are fully formed before birth, and each lobulette is seen as a little red body attached to an air-tube. By a partial or complete inflation of the fœtal lung, the arrangement of the air-sacs may be distinctly made out. The bronchial tubes at their termination have a special character. A number of alveoli, like those of the air-sacs, is found in their walls. They are best seen in the lungs of some of the lower animals, as the cat. The author has found them, in the infant, in the last divisions of the bronchial tubes and their dilated extremity; in the adult, only in the dilated extremity. They seem to become obliterated with advancing age. Their existence was first pointed out by Rossignol. The blood-vessels of the lungs: The pulmonary plexus is situated in the walls of the air-sacs; when formed, it maintains a tolerably uniform diameter throughout; the spaces between the vessels, in an injected and inflated preparation, are somewhat larger than the vessels themselves. The branches of the pulmonary artery do not anastomose till they reach the termination of the bronchial tubes; they anastomose freely in the air-sacs. The author believes that the vessels of one lobulette do not anastomose with those of another; that, consequently, in the adjoining walls of two lobulettes, two layers of capillaries lie side by side, and therefore in such situations the blood is not fully exposed to the air on both sides. The radicles of the pulmonary veins issue from the periphery of the lobulettes, and, forming larger vessels, run in the interlobular spaces to the root of the lung. After briefly alluding to the general opinion of the distribution etc. of the bronchial vessels, the author describes the result of his own injections. Injection of the pulmonary artery, so as to fill the plexus, but not the veins, does not inject the vessels of the bronchial tubes; but if the veins are filled, the bronchial tubes become partially injected. Injection of the pulmonary veins, whether the plexus be well filled or not, always injects the bronchial tubes. Injection of a bronchial artery, when fairly within the lung, produces injection of the bronchial tubes, and the fluid returns by the pulmonary veins. It is difficult, in man, to fill the vessels of the extreme bronchial tubes through the bronchial artery. The bronchial veins: The author has never been able to find the so-called deep bronchial veins as venæ-comites of the arteries. The only veins he has found have been one or two small ones, usually one, at the root of each lung, which, on being injected, were found to terminate in the structures about the root of the lung, and not to accompany the arteries within the lung. From careful injection and repeated examination of a large number of specimens, both of man and the lower animals, the author draws the following conclusions of the distribution and termination of the bronchial vessels. The bronchial arteries are distributed to the bronchi, bronchial glands, bronchial tubes, etc.—both their mucous membrane and deeper parts—the blood-vessels, and areolar tissue of the lungs; and they terminate, first, those about the root of the lung, in the bronchial veins; second, those within the lung, in the pulmonary veins. The bronchial arteries do not establish any communication with the pulmonary arteries. The author concluded by alluding to the views of previous observers.—(*Medical Times and Gazette*, July 16th, 1859.)

